# Bacterial contamination of suction catheter tips during aortic valve replacement surgery: a prospective observational cohort study

**DOI:** 10.1186/s13037-015-0066-5

**Published:** 2015-05-14

**Authors:** Johanna Larsson, Sofia Sutherland, Åsa Söderström, Christine Roman-Emanuel, Anders Jeppsson, Elisabeth Hansson Olofsson, Per-Arne Svensson

**Affiliations:** Institute of Health and Care Sciences, The Sahlgrenska Academy, University of Gothenburg, Gothenburg, Sweden; Department of Cardiothoracic Surgery, Sahlgrenska University Hospital, Gothenburg, Sweden; Department of Anaesthesia and Intensive Care, Sahlgrenska University Hospital, Gothenburg, Sweden; Department of Molecular and Clinical Medicine, Institute of Medicine, The Sahlgrenska Academy, University of Gothenburg, Vitastråket 15, 41345 Gothenburg, Sweden

**Keywords:** Suction catheter tip, Bacterial contamination, Thoracic surgery

## Abstract

**Background:**

Bacterial mediastinitis is a severe complication after open heart surgery. The infection causes prolonged hospitalization and an increased mortality risk. Observations from orthopaedic surgery showed that the suction catheter used during surgery is commonly contaminated with bacteria. The aim of this study was to describe the prevalence of suction catheter contamination in cardiac surgery and to study if suction time influences the contamination risk.

**Methods:**

Fifty suction catheter tips were collected during 25 aortic valve replacement operations. The suction tip was exchanged once during the operation (after aortotomy closure). The tips were subjected to bacterial contamination analysis.

**Results:**

In 20 of the 25 investigated cases (80%), bacterial contamination was detected on one or both tips. The tip used during the beginning of the operation showed bacterial contamination in 13/25 cases (52%) and the second tip in 12/25 (48%). In 5/25 cases (20%) both tips were contaminated. There was no association between bacterial contamination and suction time. Coagulase-negative staphylococcus was the most commonly detected microorganism.

**Conclusions:**

The suction device should be considered as a potential source of bacterial contamination in cardiac surgery. The results suggest that the suction catheter should be replaced before key moments like valve implantation and sternal closure.

## Background

Bacteria are commonly found in surgical wounds at the end of surgery [1,2]. A small quantity of bacteria with low virulence is harmless because the body’s own immunological defence can eliminate it but if the bacterial load is large it can cause surgical site infections (SSI) [3]. Bacteria may have originated from the patient’s own skin flora, from instruments or from the personnel in the operating room [3,1]. Patients who get a postoperative SSI will have a prolonged hospitalization and long-term antibiotic treatment, In addition, SSI increases healthcare expenditures [4-6].

During open-heart surgery, the surgeons do a sternotomy to gain access to the heart. In the end of the operation, sternum is closed with stainless steel wires. The steel wire remains life-long and should be viewed as an implant [7]. Infections related to foreign materials are characterized by delayed and vague symptoms, which can result in delayed diagnosis. Mediastinitis is a deep chest infection, which can emerge postoperatively after open-heart surgery. Mediastinitis affects 0.5-4% (usually 1%) of all patients who undergoes open-heart surgery and increases the mortality risk [4,8,9]. In one study, one-year mortality was 22% in patients with mediastinitis compared to 0.6% in patients without mediastinitis [4].

The colonisation of the sternotomy wound may be caused by bacteria from the patient or from the equipment or persons in the operating room [10]. The suction device is standard equipment in an operating theatre and used during most surgical procedures. Suction is used to eliminate excess fluids, for example blood. The suction device can be used with different types of suction catheters. When the suction catheter is not in use it is commonly stored in a plastic case attached to one side of the patient’s abdomen. Usually, the air flow thru the suction tip is active, even when the suction tip is not in use.

Previous research within orthopaedic surgery shows that the suction catheter tip used during surgery can be, and sometimes commonly are, contaminated with bacteria [11-17]. Contamination of suction tips was reported to be between 11-65% in these studies [11-17]. Since no data is available from cardiac surgery, the aim of this study was to investigate contamination of suction catheter tips during aortic valve replacement and to study if suction time is a factor that increases the risk of contamination.

## Methods

### Ethical considerations

No application to the Ethical Review Board was required since the study involved only surgical equipment. No identification data from the patients was collected.

### Setting

This study took place at the Department of Cardiothoracic Surgery in Sahlgrenska University Hospital, Gothenburg, Sweden. The operation ward performs approximately 1100 open-heart procedures annually. The theatres are equipped with displacement ventilation. During valve replacement surgery, a surgical team with at least six persons are present throughout the procedure.

All patients showered three times before surgery using chlorhexidine as cleaning agent and were treated with prophylactic antibiotics (three doses of cloxacillin every eight hour, the first dose administered just before the start of the operation). Skin disinfection was performed with 0,5% chlorhexidine in 70% ethanol according to routine, followed by sterile draping in a way that only exposed the surgical field. After sterile draping the medical equipment e.g. the suction device (Medela Medical AB Täby, Sweden) was connected. The disposable suction catheter was stored in a plastic case, which was attached on the right side of the patient’s abdomen. The suction catheter was connected with a tube that was attached to a canister. The canister was connected to a wall suction system. The amount of air drawn through the suction catheter is 50 litres/minute. A disposable Argyle™ flexible yankauer suction catheter (Covidien, Dublin, Ireland) was used.

### Sampling method

Suction catheter tips were collected during March-May 2014 on 25 valve replacement surgeries on adults without on-going infection. The suction device aspirated air throughout the surgery. From each surgery, two suction catheters were collected. The first suction catheter was used until and during closure of the aortotomy. The second suction catheter was used after the closure of the aortotomy and until the end of the operation. A research nurse noted the time for surgery start, suction catheter exchange and surgery end. The distal three centimetres of each suction catheter tip were cut off with a sterile pair of nippers and placed in a sterile container. Five unused control suction catheters were also included in the analysis. The control suction catheters were connected to the suction device according to the manual, and were cut off and collected before the surgery started. The containers with the suction tips were stored according to the routines of the ward and then sent for contamination analysis.

### Contamination analysis

The suction catheter tips were analysed at the Department of Microbiology at Sahlgrenska University Hospital (accredited by Swedac). The tips were rolled on aerobe and anaerobe horse blood agar, Drigalski agar and Grand Lux plate (nutritional medium used for bacterial culturing). The blood agar and the Grand Lux plate were incubated in CO_2_ in 35°C for four days. The Drigalski agar was incubated in ordinary indoor air in 35°C. The suction tips were then put into tubes with Thioglycollate broth and incubated for two days in 35°C and the broth was then subculture onto aerobe and anaerobe horse blood agar and incubated in CO_2_ at 35°C for two days. This procedure is referred to as the enrichment procedure. Bacterial contamination was classified into four categories based on numbers of colony forming units (CFU); abundant (>100 CFU), moderate (10–100 CFU), sparse (<10 CFU) or after enrichment. Bacterial isolates were identified by standard microbiological techniques

### Data analysis

Statistical analysis was performed using SPSS 22. Data was analysed using Chi-square or Fishers’ exact test (where appropriate). Frequencies of contamination were compared between the first part and the second suction tips. Frequencies of contamination were also compared within first part and second part of the operation, using time stratified data (above or below median operation time).

## Results

### Contamination

Bacterial contamination was observed in one or both of the suction catheter tips in 20/25 cases (80%) of the studied operations. In 5/25 cases (20%), both the first and the second tip were contaminated. None of the control suction catheter tips were contaminated.

From the first part of the operation (before aortotomy closure), 13 out of the 25 suction catheter tips (52%) showed bacterial contamination. The median time for the first part of the operation was 103 minutes (range 60–138 minutes). From the second part of the operation (after aortotomy closure), 12 out of 25 suction catheter tips (48%) were contaminated. The median time of the second part of the operation was 74 minutes (range 50–187 minutes). There were no significant difference in contamination frequencies between the first and the second part of the operation (*p*-value 0.32).

Our hypothesis that time may be an important factor for contamination was analysed using operation time stratified data. During the first part of the operation, the short time was defined as between 60–100 minutes and the long time was defined as between 105–138 minutes. The corresponding times for the second part of the operation were between 50–68 minutes for short and between 78–187 minutes for long time. There were no significant differences in contamination frequencies between short and long operation time, neither during the first nor during the second part of the operations (*p*-value 0.68 and 0.68, respectively).

### Quantification of bacterial contaminants

**Figure 1:**
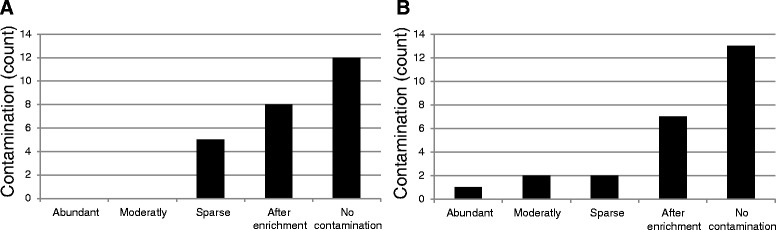
Quantification of bacterial growth on suction catheter tips from the first part **(A)** and the second part **(B)** the aortic valve replacement operations. Bacterial contamination was classified into four categories; abundant (>100 CFU), moderate (10–100 CFU), sparse (<10 CFU) or after enrichment.

Quantification of the contamination of the suction catheters tips are shown in Figure 1. From the first part of the operation, five samples showed sparse bacterial contamination and eight showed bacterial contamination after enrichment (Figure 1A). From the second part of the operation, one sample showed abundant growth, two samples moderate growth, two showed sparse growth and seven samples showed bacterial growth after enrichment (Figure 1B). If samples where the detection was observed only after enrichment were excluded from the analysis, contamination frequencies of the catheter suction tips were 20% for both the first part and the second part of the operations.

### Species classification of bacterial contaminants

**Figure 2:**
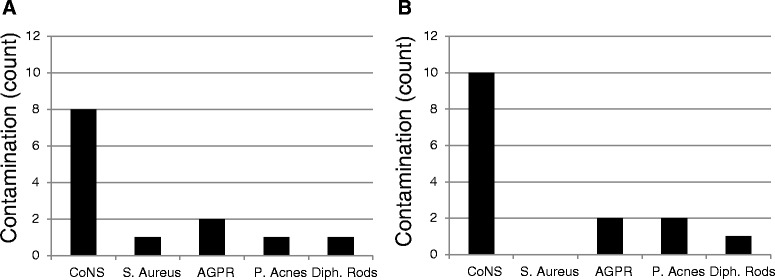
Identification of the suction catheter tip contaminating bacteria for the first part **(A)** and the second part **(B)** of the aortic valve replacement operations. If a suction catheter tip was contaminated by more than one species of bacteria, they are represented as multiple counts in the figure. CoNS; coagulase-negative staphylococci. *S. Aureus; Staphylococcus Aureus*, AGPR; Anaerobic gram-positive rods, *P. Acnes; Propionibacterium Acnes*, Diph. Rods; Diphteroid gram-positive rods.

From the 13 contaminated suction catheter tips from the first part of the operation, only one type of bacteria was identified on each suction tip (Figure 2A). Among the 12 contaminated suction catheter tips from the second part of the operation, two different bacterial species were identified in the culture in three cases (Figure 2B).

The most common bacteria that were cultured were coagulase-negative staphylococci. It was found in eight cases from the first part of the operation and in ten cases from the second part (Figure 2A and 2B). *Staphylococcus Aureus* was shown only in one case overall (cultured in the first part). Anaerobic gram-positive rods were found in two cases from each part of the operations. *Propionibacterium Acnes* were cultured in one case from the first part and two cases from the second part. From each part of the operations, one case of Diphteroid gram-positive rods was found.

## Discussion

In our study, bacterial contamination was detected in 80% in one or both of the suction catheter tips and that contamination can be detected both during and in the end of the operation. We found no evidence supporting the hypothesis that suction time is a predictor of suction catheter tip contamination. Despite advances in operating theatre design aimed at reducing contamination risk, the suction catheter tip should be considered as a potential source of bacterial contamination of the surgical wound.

From the first part of the operations the suction catheter tips were contaminated in 52% of the cases. From the second part of surgery 48% were contaminated. These amounts of contaminated samples match findings from previous studies in other types of operations [12,14,17]. If we consider bacterial contamination in ether the first or second suction catheter tips, the frequencies observed in this study (80%) surpasses frequencies observed in previous studies [11-17]. However, there are methodological differences in how the bacterial culturing was performed and not all studies used methods equivalent to the enrichment procedure used in our study. This procedure most likely improves contamination detection since it also enables the detection of contaminates that have been sucked into the tip and attaches to the inside the suction tip. If contamination detection after enrichment is not included in the analysis, contamination frequencies are reduced to 20%. Since the contamination frequency was high (regardless if considering 80% or 20% as the contamination frequency) it is not illogical to suggest that the suction catheter should be exchanged before key procedures, such as when the aortotomy is sutured and before sternum is closed with the stainless steel wires. Especially since the cost of a suction catheter is very low and the changing procedure is very easy and not time consuming.

In previous studies, suction time has been shown to be related to bacterial contamination [12,13]. In our study no difference in bacterial contamination between different surgical times was observed. It is logical to assume that surgical time, and hence also the volume of air sucked into the suction catheter tip, could be related to contamination levels. It is unclear why such relationship was not observed in this study since both contamination frequencies and surgical times were in the same range as previous studies showing such relationships [12,13].

Coagulase-negative staphylococci is the most common bacteria cultured on suction catheters according to several studies [11-17]. Coagulase-negative staphylococci is also known as one of the most common bacteria causing mediastinitis [8,18] Our findings with high frequencies of coagulase-negative staphylococci contamination of the suction tips are in line with previous studies but also worrying from a mediastinitis point of view. Other bacteria that were found were *Staphylococcus aureus*, Anaerobe gram-positive rods, *Propionibacterium acnes* and Diphteroid rods. However, these bacterial species were always found less frequently. Since both coagulase-negative staphylococci and *Staphylococcus aureus* are part of the normal skin flora, these contaminations may be derived from the patient or from the staff in the operating room [3]. Such results indicate that care must be taken by every individual in the operation room to reduce the risk of contamination.

The control suction catheter tips were all negative for bacterial growth. This indicates that the handling of the suction catheter before surgery, collection of the samples and the procedure at the microbiology department did not frequently contaminate the suction catheter tips. Limitations of the study are that it includes only a limited number of observations from a single hospital. The analysis of the impact of suction time on contamination frequencies is limited also by homogeneity in the operation times. The results from the study is in line with previous older studies of this topic and can therefore probably be generalized to surgeries of similar length, at operating theatres with similar ventilation and similar use of the suction catheter tip.

## Conclusion

This study shows that the suction catheter tips should be considered as a potential source of bacterial contamination of the surgical wound. We suggest that the suction catheter should be replaced during the operation and before critical moments, like when the sternum is closed. Due to the low cost of a suction catheter compared to the cost of treating a patient with mediastinitis it is justified from an economical point of view. However, large randomized trials with postoperative wound infections as endpoints are needed to determine if frequent suction catheter exchanges is a truly effective improvement.
